# Molecular distribution and isotopic composition (δD and δ^13^C) of lipids dataset for a sediment core (GeoB16202-2) from the north-eastern Brazilian margin

**DOI:** 10.1016/j.dib.2020.106508

**Published:** 2020-11-06

**Authors:** Laura Rozo, Leticia Lazzari, Enno Schefuß, Stefan Mulitza, Renato Carreira

**Affiliations:** a LabMAM, Chemistry Department, Pontifical Catholic University of Rio de Janeiro (PUC-Rio), 22451-900 Rio de Janeiro, Brazil; b MARUM-Center for Marine Environmental Sciences, University of Bremen, D-28359 Bremen, Germany

**Keywords:** Lipids biomarkers, Paleoclimate, Stable carbon and hydrogen isotopes, Heinrich Stadial 1, Continental precipitation

## Abstract

The distribution and isotopic composition (δ^13^C and δD) of lipids are proxies used to determinate paleoenvironmental conditions including precipitation regimes, vegetation changes and sources of organic matter, among others. This data article describes five datasets of distribution (*n*-alkanes, fatty acids, *n*-alkanols and sterols) and isotopic composition (*n*-alkanes and fatty acids) of lipids determined in 50 samples from a gravity core (GeoB16202–2) retrieved on the continental slope off northeastern Brazil. The core site is influenced by the Parnaiba freshwater discharge, the North Brazil Current and by the Intertropical Convergence Zone (ITCZ). Previous work focused on inorganic proxies in this core revealed important clues of climatic conditions during the Heinrich Stadial 1 (HS1). The baseline dataset of molecular and isotopic proxies of the organic matter provided here are additional and/or complimentary evidences to help elucidate past climate change during the Quaternary in the Equatorial Atlantic, where less information is available in comparison to other regions in this ocean.

## Specifications Table

**Table:** 

Subject	Environmental Sciences
Specific subject area	Organic Geochemistry for paleoclimate and environmental studies.
Type of data	Table-as supplementary material Figure
How data were acquired	Analytical Instrumentation: GC-IRMS system (Gas Chromatograph model Trace Ultra coupled to mass spectrometer MAT252 from Thermo Scientific -for carbon isotope determination- and Gas Chromatograph model Trace Ultra coupled to mass spectrometer MAT253 from Thermo Scientific -for hydrogen isotope determination-) (Detection Limit 100 ng/g); Gas Chromatograph with Flame Ionization Detector GC-FID model Focus from Thermo Finnigan to determinate the distribution of *n*-alkanes and fatty acids (Detection Limit 2 ng/g); Gas Chromatograph coupled to mass spectrometer GC/MS model DSQ from Thermo Finnigan for *n*-alkanols and sterols analysis (Detection Limit 1 ng/g). Software: QGIS (version 3.10.4), Grapher (Version 15.3).
Data format	Raw Analyzed and calculated.
Parameters for data collection	Sediments samples (*n* = 50) were collected from core GeoB16202–2 (773 cm) in order to achieve temporal coverage of molecular distribution and isotopic composition (δ_D_ and δ^13^C of lipids biomarkers) data in the continental slope off northeastern Brazil.
Description of data collection	The sediment core covers the Last Glacial Maximun (LGM), including millenial cold events as Heinrich Stadial 1 (HS1), Younger Dryas (YD) and the warm Bolling-Allerod interstadial. Lipids (*n*-alkanes, *n-*fatty acids, *n*-alkanols and sterols) were extracted from sediments in an ASE (Accelerated solvent extractor), purified by column chromatography with silica and identified/quantified by GC-FID and GC/MS intrumental analyses. The isotopic composition of carbon and hydrogen of the terrigenous markers (long-chain *n*-alkanes and fatty acids) were determined by GC-IRMS. Selected indexes based on the lipid biomarkers – average chain lenght (ACL) and carbon preference index (CPI),– were calculated to evaluate the relative contribution of different sources of organic matter to the sediment.
Data source location	Institution: MARUM/University of Bremen and Pontifical Catholic University City/Town/Region: Northeast Brazil (Barreirinhas/Ceara Basin) Country: Brazil Latitude and longitude (and GPS coordinates) for collected samples/data: Gravity core GeoB16202–2 (1°54.50′S, 41°35.50′W)
Data accessibility	The raw data is available as a supplementary data file to this article.

## Value of the Data

• This dataset is useful to elucidate the nature and distribution of the lipids accumulated during the last 23 ka BP on the core site. This accumulation can be corelated with different climatic process and with other paleoenvironmental proxies.
• This data can be used by other researchers to model climatic process that are important in this area as well to compare with other records with similar molecular and/or compound-specific isotopic composition record.
• The molecular distribution and isotopic composition data have an important role to understand the sources of organic matter accumulated in the core. The lipid biomarkers characterize autochthonous and allochthonous sources of organic matter to the study site, whereas the carbon isotope composition of lipids can indicate changes in vegetation and the hydrogen isotope composition has been shown a good proxy for hydrological regimes.

## 1 Data Description

**Fig. 1:**
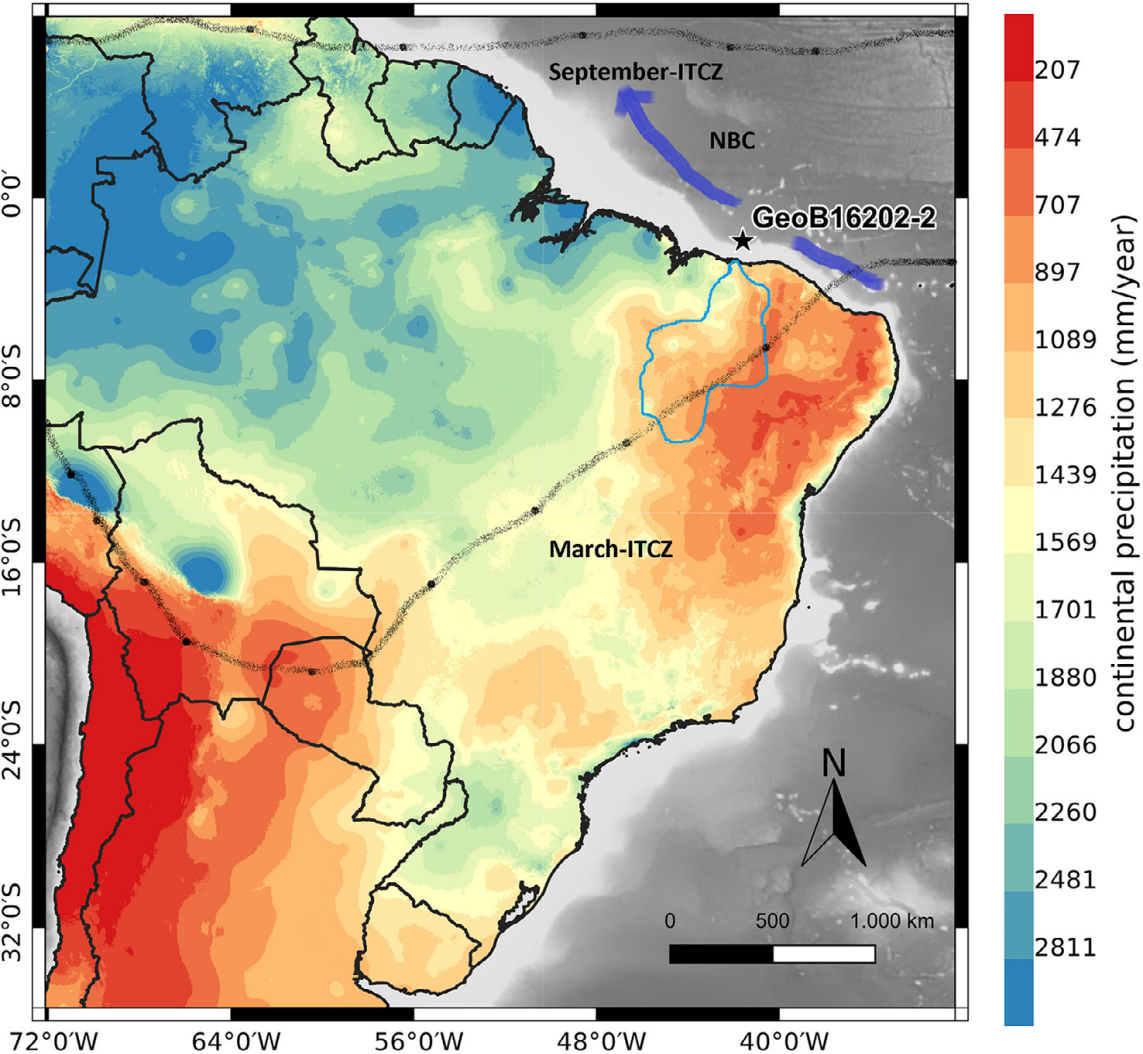
Location of site GeoB16202–2, modern continental precipitation (mm/year) for South America and the Parnaiba catchment region (blue line) [1]. The North Brazil Current (NBC) flowing to the NW is also indicated and the modern seasonal position for the ITCZ.

**Fig. 2:**
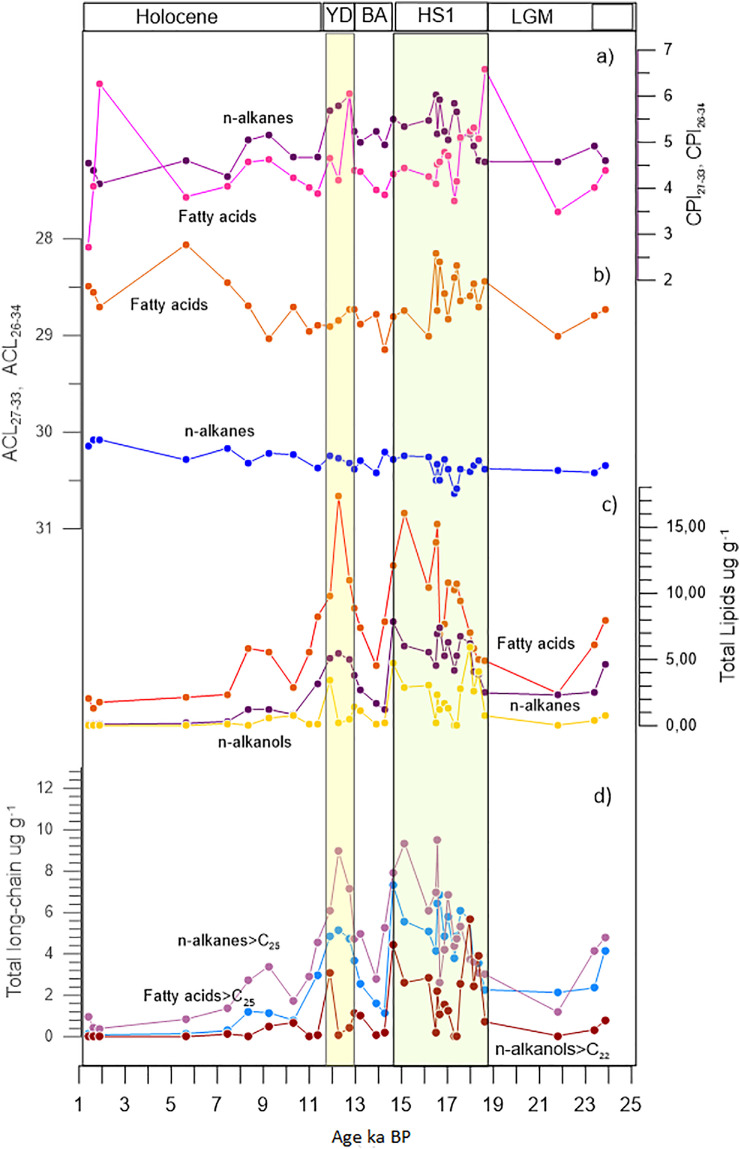
Downcore variability of n-alkyl lipids during last 23 kyr covering Last Glacial Maximum (LGM), Heinrich Stadial 1 (HS1), Bolling-Allerod (BA), Younger Dryas (YD) and Holocene periods. a) Carbon Preference Index (CPI) for n-alkanes (purple) and fatty acids (pink). b) Average chain length index (ACL) for *n*-alkanes (blue) and fatty acids (orange). c) Total lipids, *n*-alkanes (purple), fatty acids (red), *n*-alkanols (yellow). d) Total long-chain lipids, n-alkanes (blue), fatty acids (purple), n-alkanols (red).

**Fig. 3:**
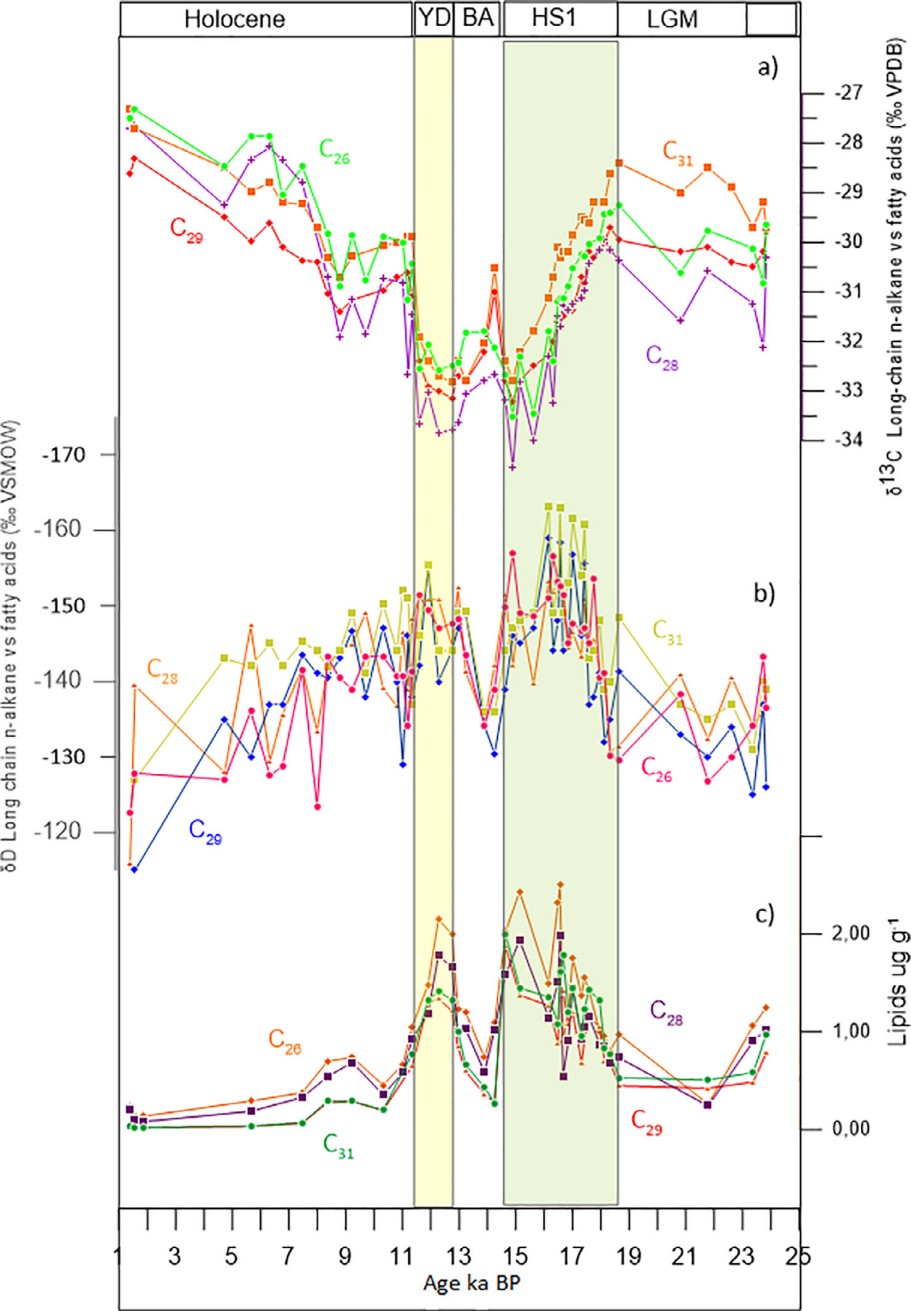
Downcore variability of isotopic composition (δD, δ^13^C) for long-chain lipids. a) δ^13^C of n-alkanes (C_29_ red, C_31_ orange) and fatty acids (C_26_ green, C_28_ purple). b) δD of *n*-alkanes (C_29_ blue, C_31_ ligth green) and fatty acids (C_26_ pink, C_28_ orange). c) Total long-chain *n*-alkanes (C_29_ red, C_31_ green), and fatty acids (C_26_ orange, C_28_ purple).

Fig. 1 shows the location of sediment core GeoB16202–2 as well as the modern continental precipitation and the Parnaiba River catchment area. Table 1, Table 2, Table 3, Table 4 present, respectively, the molecular distribution of *n*-alkanes, fatty acids, *n***-**alkanols and sterols, obtained in 35 samples analyzed from the sediment core GeoB16202–2. Table 5 shows the carbon and hydrogen isotopic composition of long-chain *n*-alkanes (C_29_, C_31_) and long-chain fatty acids (C_26_, C_28_) determined in 50 samples from core GeoB16202–2. Fig. 2 shows the temporal distribution of lipids (*n*-alkanes, fatty acids and *n*-alkanols) and the variations in the values of the Carbon Preference Index (CPI) and the Average Chain Length (ACL). The CPI is a molecular ratio reflecting the relative distribution of odd- and even- carbon chain among the *n*-alkanes and *n*-fatty acids that reflects the organic matter source and/or alteration and can be related to paleoenvironmental conditions. The ACL reflects the average carbon chain length of plant leaf waxes, and has been widely used as indicator of land vegetation and environmental conditions because woody plants has lower ACL values compared to non-woody plants. Fig. 3 shows the temporal variation of carbon isotopic composition (δ^13^C) and hydrogen isotopic composition (δD) for long-chain *n*-alkanes (C_29_ and C_31_) and long-chain fatty acids (C_26_ and C_28_) and shows the change in the concentration of these compounds in the last 23 ka BP.

**Table 1:** Molecular distribution of *n*-alkanes C_19_—C_35_ in ng g^−1^ in the sediment core GeoB16202–2.

Depth (m)	Age (ka)	nC_19_	nC_20_	nC_21_	nC_22_	nC_23_	nC_24_	nC_25_	nC_26_	nC_27_	nC_28_	nC_29_	nC_30_	nC_31_	nC_32_	nC_33_	nC_34_	nC_35_	Total	Tot.>C_25–35_	ACL_27–33_	CPI_25–35_	CPI_27–33_
0.08	1.39	1.33	2.62	2.33	2.66	4.31	3.51	6.68	4.61	12.6	5.89	27.3	5.95	30.4	4.40	17.6	1.73	1.33	135	118	30.15	2.12	4.55
0.11	1.57	0.97	1.88	1.87	2.18	3.42	2.73	4.87	3.17	8.21	3.76	16.4	3.47	17.6	2.70	10.1	1.07	1.17	86	73	30.08	2.06	4.38
0.14	1.85	0.93	2.29	1.74	2.13	3.67	3.23	5.62	3.93	9.85	4.80	19.9	4.53	21.6	3.51	12.2	1.58	3.44	105	91	30.08	1.98	4.10
0.44	5.65	1.31	3.13	2.63	2.77	5.10	4.10	8.47	5.20	14.2	6.55	32.5	7.26	37.6	6.17	25.1	2.48	7.95	172	153	30.29	2.27	4.61
0.79	7.47	3.06	3.46	5.54	5.64	11.4	8.14	18.2	9.95	30.5	13.5	65.1	14.9	68.9	12.7	43.4	5.48	14.7	335	297	30.16	2.13	4.27
0.99	8.36	<DL	<DL	<DL	<DL	22.8	15.6	58.6	24.1	110	43.2	276	56.9	293	51.9	207	23.9	75.4	1259	1220	30.32	2.55	5.04
1.22	9.23	4.00	4.54	10.1	10.3	26.7	16.6	57.0	23.7	107	43.5	288	54.9	290	47.9	177	18.0	56.2	1237	1164	30.22	2.60	5.16
1.49	10.3	5.38	4.76	8.34	8.51	19.4	12.8	36.8	18.0	72.6	30.9	195	37.9	200	33.0	124	32.8	12.9	853	794	30.23	2.10	4.67
1.80	11.3	10.8	13.0	27.0	32.0	78.0	51.4	129	71.8	243	115	646	148	773	142	504	48.7	151	3184	2972	30.38	2.33	4.67
2.04	11.9	11.0	13.4	37.5	42.5	120	67.1	205	88.7	398	162	1288	227	1320	195	723	57.1	188	5145	4852	30.25	2.83	5.69
2.25	12.3	8.21	11.8	34.4	41.5	125	70.2	225	94.7	420	168	1340	232	1418	206	786	63.3	203	5447	5156	30.27	2.87	5.79
2.52	12.7	8.39	10.0	31.0	35.6	113	61.7	208	86.1	369	156	1215	205	1326	182	766	50.8	179	5001	4741	30.32	2.99	6.03
2.65	13.0	4.84	6.52	20.1	26.2	83.0	47.7	153	67.3	289	125	842	170	995	168	622	56.7	169	3846	3658	30.38	2.62	5.23
2.78	13.2	5.80	6.80	18.6	21.3	63.3	36.6	120	52.9	224	94.9	596	123	667	115	406	36.6	100	2688	2536	30.30	2.50	5.01
2.98	13.9	4.44	4.93	12.1	14.2	36.3	23.8	67.8	34.5	123	58.5	361	73.8	430	69.3	289	22.7	77.7	1703	1608	30.42	2.61	5.23
3.09	14.3	<DL	<DL	7.09	7.80	21.7	16.3	50.6	27.7	110	45.8	280	55.4	273	47.4	182	17.8	61.0	1204	1151	30.21	2.46	4.94
3.25	14.6	13.8	18.2	61.3	77.5	212	117	320	147	596	249	1871	339	1999	306	1121	91.5	303	7843	7343	30.28	2.74	5.51
3.50	15.1	11.2	16.4	60.2	77.6	208	109	289	125	496	199	1376	254	1449	231	850	70.2	236	6059	5576	30.25	2.67	5.35
4.04	16.2	9.07	14.5	59.5	75.9	213	105	291	117	491	183	1266	228	1357	213	826	73.7	39.5	5561	5084	30.25	2.62	5.49
4.40	16.5	7.62	12.0	51.4	60.1	169	76.2	206	78.1	319	121	875	158	1085	162	800	59.8	275	4516	4139	30.49	3.07	6.03
4.48	16.6	10.2	16.6	65.0	86.7	242	126	348	146	579	224	1406	285	1618	273	1077	98.4	366	6967	6420	30.33	2.63	5.18
4.60	16.7	10.8	15.8	66.1	85.0	243	119	336	134	534	207	1417	268	1786	265	1339	102	464	7392	6852	30.50	3.01	5.92
4.80	16.9	9.28	14.3	58.4	74.7	211	103	286	113	462	170	1079	213	1207	201	783	69.5	259	5314	4844	30.28	2.66	5.23
4.98	17.0	9.08	14.5	57.2	76.8	219	114	315	132	519	200	1199	258	1448	257	1019	97.4	363	6298	5809	30.38	2.57	5.04
5.35	17.3	6.18	10.7	49.6	60.2	165	73.6	198	73.0	284	104	681	138	955	156	821	67.8	323	4168	3803	30.63	3.02	5.84
5.48	17.4	6.67	10.5	44.9	57.5	174	83.3	239	95.7	376	144	905	189	1231	202	1021	84.5	396	5260	4883	30.58	2.91	5.65
5.68	17.6	10.7	18.4	83.2	101	294	138	378	149	598	206	1157	252	1429	259	1108	110	424	6716	6072	30.38	2.61	5.07
6.12	18.0	8.95	15.0	69.5	85.4	254	123	346	136	558	186	1049	228	1322	235	1062	109	450	6237	5682	30.41	2.67	5.17
6.30	18.1	7.34	11.7	47.4	57.9	170	84.8	235	94.9	375	130	691	155	840	156	658	66.8	262	4042	3663	30.35	2.54	4.92
6.50	18.3	6.82	10.8	44.7	58.2	168	90.0	239	100	371	139	686	157	774	154	610	64.4	244	3917	3538	30.29	2.38	4.59
6.74	18.6	6.69	8.76	25.8	34.3	87.5	54.0	129	64.3	201	91.5	449	107	532	98.2	406	36.9	151	2483	2266	30.38	2.35	4.58
7.25	21.8	6.80	8.86	23.5	31.1	63.5	50.1	115	60.3	183	86.6	422	102	508	92.2	388	35.3	145	2321	2137	30.40	2.34	4.57
7.50	23.4	7.00	8.85	23.0	28.9	73.8	46.4	115	60.0	193	90.2	488	108	585	98.5	431	37.2	152	2546	2358	30.42	2.49	4.91
7.60	23.8	9.16	14.9	56.7	73.3	196	103	266	115	418	156	786	191	976	191	722	73.7	271	4618	4165	30.34	2.37	4.60
Min.		1	2	2	2	3	3	5	3	8	4	16	3	18	3	10	1	1	86	73	30.08	1.98	4.10
Max.		14	18	83	101	294	138	378	149	598	249	1871	339	1999	306	1339	110	464	7843	7343	30.63	3.07	6.03
Average		7	10	35	44	121	63	177	75	297	117	744	149	861	142	586	53	189	3666	3389	30.32	2.55	5.09

ACL_27–33_ = (Σ n C_n_ / Σ C_n_); CPI_25–35_= Σ (C_25_ + C_27_ +C_29_ + C_31_ +C_33_ + C_35_)/(2 Σ (C_26_ + C_28_ +C_30_ + C_32_ +C_34_)); CPI_27–33_= (Σ (C_27_–_33_)/Σ (C_26–32_)) +(Σ (C_27_–_33_)/Σ (C_28_–_34_))*0.5; Paq = (C_23_ + C_25_)/(C_23_ + C_25_ +C_29_ + C_31_); DL=detection limit.

**Table 2:** Molecular distribution of n-fatty acids C_16_—C_34_ in ng g^−1^ in the sediment core GeoB16202–2.

Depth (m)	Age (ka)	*n*C_16_	*n*C_18_	*n*C_19_	*n*C_20_	*n*C_21_	*n*C_22_	*n*C_23_	*n*C_24_	*n*C_25_	*n*C_26_	*n*C_27_	*n*C_28_	*n*C_29_	*n*C_30_	*n*C_31_	*n*C_32_	*n*C_33_	*n*C_34_	Total	CPIC_26–32_	ACL C_26–34_	CPIC_20–32_	ACLC_20–34_
0.08	1.39	290	222	<DL	73	<DL	60	245	205	97	228	105	205	85	117	<DL	72	<DL	63	2066	2.72	28.49	1.81	26.16
0.11	1.57	276	179	19	45	19	44	134	138	36	143	39	96	21	37	<DL	22	<DL	62	1310	4.04	28.56	2.03	25.78
0.14	1.85	305	223	55	122	55	68	396	148	36	136	13	81	13	31	<DL	22	7	53	1772	6.26	28.70	1.10	24.48
0.44	5.65	395	228	34	73	34	56	253	234	75	292	86	187	37	64	<DL	37	<DL	66	2151	3.82	28.05	1.88	25.64
0.79	7.47	315	278	21	55	24	42	39	210	72	378	97	328	73	194	43	136	20	37	2360	4.04	28.46	3.65	27.13
0.99	8.36	835	438	132	219	80	61	833	536	150	700	145	543	144	495	103	339	<DL	86	5839	4.57	28.69	1.91	26.27
1.22	9.23	824	424	42	208	60	<DL	111	528	141	739	156	687	175	682	130	490	67	131	5593	4.62	29.04	4.03	27.58
1.49	10.32	376	280	23	80	27	59	52	258	85	457	100	354	94	305	63	205	31	55	2904	4.23	28.71	3.84	27.30
1.68	11.01	539	368	64	209	62	<DL	919	458	141	666	162	582	164	537	113	384	65	121	5553	4.02	28.95	1.74	26.63
1.80	11.34	1026	609	80	366	99	<DL	715	740	240	1051	268	926	263	827	174	562	93	178	8217	3.89	28.89	2.42	26.92
2.04	11.90	1078	575	59	302	88	<DL	244	1379	353	1484	286	1188	273	1108	201	796	120	254	9789	4.66	28.90	4.08	27.30
2.25	12.27	2610	1443	141	948	229	166	547	2305	531	2155	468	1782	469	1641	333	1092	159	326	17,344	4.19	28.84	3.70	26.73
2.52	12.74	1304	657	64	178	91	42	56	1441	122	1997	99	1660	440	1374	293	769	131	237	10,956	6.05	28.73	6.23	27.60
2.65	12.96	1231	716	62	502	96	<DL	340	1132	267	1237	250	999	236	804	156	529	91	192	8838	4.40	28.74	3.66	26.77
2.78	13.23	633	466	41	181	59	<DL	144	847	240	1202	245	1028	260	912	185	613	109	199	7365	4.37	28.88	3.96	27.60
2.98	13.90	563	358	<DL	62	51	62	109	531	21	735	184	587	181	483	128	321	66	115	4557	3.97	28.78	3.94	27.51
3.09	14.25	691	545	50	256	72	45	164	783	252	1089	277	1022	286	935	223	744	144	278	7857	3.86	29.15	3.56	27.73
3.25	14.63	1057	552	57	390	103	<DL	323	1717	494	2025	415	1586	375	1304	261	951	160	345	12,115	4.32	28.80	3.83	27.25
3.50	15.14	1759	1034	97	935	184	<DL	470	2197	571	2437	484	1937	442	1564	311	1075	168	368	16,034	4.43	28.74	3.91	26.85
4.04	16.16	1521	528	41	438	84	36	257	1400	343	1498	262	1143	252	1057	281	804	190	283	10,417	4.27	29.01	4.01	27.23
4.40	16.49	1678	824	97	993	203	<DL	570	2484	589	2326	424	1508	307	896	261	464	65	156	13,846	4.10	28.14	3.56	25.96
4.48	16.56	1341	782	83	845	159	<DL	420	2099	552	2507	484	1989	434	1532	311	1077	185	408	15,207	4.51	28.74	4.03	26.97
4.60	16.67	1171	652	140	702	144	<DL	274	1217	125	895	161	543	125	354	102	230	<DL	79	6914	4.58	28.23	3.95	25.44
4.80	16.85	1089	464	40	462	78	29	220	1098	276	1198	214	911	180	677	127	446	66	139	7714	4.78	28.57	4.20	26.61
4.98	17.01	1239	522	47	411	85	58	255	1369	366	1758	329	1451	304	1127	226	840	144	319	10,850	4.69	28.83	4.23	27.26
5.35	17.32	1536	859	93	914	154	146	493	1695	290	1371	283	928	131	542	277	362	73	133	10,279	3.73	28.40	3.48	25.72
5.48	17.42	1474	832	97	949	171	159	434	1850	213	1556	360	1045	162	601	197	374	79	143	10,696	4.16	28.27	4.02	25.74
5.68	17.58	1125	560	51	504	103	<DL	301	1462	368	1427	266	1163	224	867	164	609	<DL	230	9425	5.10	28.64	4.16	26.69
6.12	17.96	799	565	40	565	78	<DL	206	1068	247	1007	183	872	159	613	114	402	<DL	154	7072	5.23	28.59	4.49	26.40
6.30	18.13	447	315	27	286	53	42	155	869	221	956	176	852	162	643	121	466	<DL	<DL	5791	5.32	28.47	4.56	26.75
6.50	18.33	437	301	23	215	39	13	123	747	199	809	154	678	133	500	97	368	<DL	150	4987	5.07	28.70	4.39	26.99
6.74	18.64	595	243	<DL	129	45	<DL	136	746	95	972	101	734	108	477	94	287	52	96	4910	6.58	28.44	5.54	27.01
7.25	21.79	399	216	26	210	39	52	59	255	67	263	69	243	71	192	55	148	31	61	2453	3.49	29.00	3.52	26.54
7.50	23.38	631	275	<DL	171	47	42	125	712	29	1064	265	903	268	680	186	483	96	178	6157	4.01	28.79	4.20	27.57
7.60	23.84	760	478	57	437	88	<DL	228	1109	305	1249	248	1024	215	744	160	536	97	203	7939	4.38	28.73	3.86	26.90
Min.		276	179	19	45	19	13	39	138	21	136	13	81	13	31	43	22	7	37	1310	2.72	28.05	1.10	24.48
Max.		2610	1443	141	993	229	166	919	2484	589	2507	484	1989	469	1641	333	1092	190	408	17,344	6.58	29.15	6.23	27.73
Average		924	515	61	384	88	64	296	1028	235	1143	225	908	208	712	177	487	96	173	7637	4.47	28.68	3.64	26.72

ACL_26–34_ = (Σ n C_n_ / Σ C_n_); ACL_20–34_ = (Σ n C_n_ / Σ C_n_); CPI_20–32_= (Σ (C_20_–_32_)/Σ (C_19–31_)) +(Σ (C_20_–_32_)/Σ (C_21_–_33_))*0.5; CPI_26–32_= (Σ (C_26_–_32_)/ Σ (C_25–31_)) +(Σ (C_26_–_32_)/Σ (C_27_–_33_))*0.5; DL=detection limit.

**Table 3:** Molecular Distribution of *n*-alkanols C_14_—C_32_ in ngg^−1^ from sediment core GeoB16202–2.

Depth (m)	Age (ka)	C_14_—OH	C_16_—OH	C_18_—OH	C_20_—OH	C_22_—OH	C_24_—OH	C_25_—OH	C_26_—OH	C_27_—OH	C_28_—OH	C_29_—OH	C_30_—OH	C_31_—OH	C_32_—OH	Total	Tot.>C_22_	Tot.<C_22_	ACL _22–32_
0.08	1.39	<DL	<DL	<DL	<DL	<DL	<DL	<DL	<DL	<DL	<DL	<DL	<DL	<DL	<DL	<DL	<DL	<DL	–
0.11	1.57	<DL	<DL	<DL	<DL	<DL	<DL	<DL	<DL	<DL	<DL	<DL	<DL	<DL	<DL	<DL	<DL	<DL	–
0.14	1.85	<DL	<DL	<DL	<DL	<DL	<DL	<DL	<DL	8	<DL	<DL	<DL	<DL	<DL	8	8	<DL	27.00
0.44	5.65	<DL	<DL	<DL	<DL	<DL	<DL	<DL	<DL	<DL	<DL	<DL	<DL	<DL	<DL	<DL	<DL	<DL	–
0.79	7.47	<DL	10	<DL	38	21	29	<DL	12	<DL	18	<DL	19	<DL	21	169	120	49	26.82
0.99	8.36	<DL	<DL	<DL	<DL	<DL	7	<DL	8	<DL	13	<DL	9	<DL	7	45	45	0	28.06
1.22	9.23	13	27	9	11	39	86	13	63	<DL	110	12	106	15	73	578	517	61	27.65
1.49	10.32	8	21	12	25	87	135	14	79	15	130	14	120	8	83	752	687	66	27.03
1.68	11.01	9	20	16	16	19	15	<DL	<DL	<DL	<DL	<DL	<DL	<DL	<DL	95	34	61	22.89
1.80	11.34	<DL	<DL	8	9	<DL	12	<DL	8	<DL	20	<DL	17	8	23	104	88	17	29.00
2.04	11.90	30	84	85	124	656	676	129	507	65	464	64	271	25	216	3396	3073	323	25.86
2.25	12.27	18	19	24	17	31	28	<DL	26	<DL	8	<DL	10	10	<DL	189	112	78	25.33
2.52	12.74	10	<DL	11	15	38	53	13	69	8	96	12	86	<DL	62	472	437	35	27.57
2.65	12.96	25	73	87	92	298	260	42	218	20	97	22	100	12	67	1413	1136	277	25.46
2.78	13.23	7	26	20	29	182	200	28	173	27	154	26	126	10	103	1111	1028	83	26.41
2.98	13.90	8	<DL	<DL	8	11	46	<DL	15	<DL	17	<DL	8	<DL	<DL	112	96	16	25.28
3.09	14.25	<DL	<DL	<DL	7	18	25	<DL	24	<DL	37	<DL	36	<DL	34	181	174	7	27.72
3.25	14.63	36	56	77	105	520	788	188	826	166	798	128	478	88	457	4711	4437	274	26.77
3.50	15.14	20	83	94	109	386	471	125	447	115	440	55	266	38	277	2925	2619	307	26.56
4.04	16.16	11	44	46	100	521	636	150	568	92	171	66	266	60	304	3035	2834	201	26.11
4.40	16.49	<DL	10	15	15	50	42	12	36	<DL	36	<DL	16	<DL	16	248	208	40	25.69
4.48	16.56	14	28	24	53	243	369	136	398	80	391	56	215	45	251	2302	2184	118	26.81
4.60	16.67	14	21	20	44	209	200	61	195	21	187	17	41	16	156	1202	1103	99	26.30
4.80	16.85	13	35	30	55	258	307	98	270	48	238	34	120	22	148	1677	1544	133	26.24
4.98	17.01	<DL	11	18	21	156	177	36	194	41	227	25	162	26	202	1295	1245	50	27.24
5.35	17.32	<DL	<DL	9	8	<DL	<DL	<DL	<DL	<DL	<DL	<DL	17	15	9	58	41	16	30.81
5.48	17.42	<DL	<DL	<DL	<DL	<DL	<DL	<DL	<DL	<DL	<DL	<DL	<DL	<DL	<DL	<DL	<DL	<DL	–
5.68	17.58	20	66	39	101	436	368	141	398	99	446	74	213	55	323	2779	2553	226	26.65
6.12	17.96	12	76	51	111	778	855	320	1004	213	1140	140	487	104	657	5946	5697	250	26.73
6.30	18.13	17	32	25	93	474	397	144	391	109	407	51	180	30	249	2597	2431	167	26.25
6.50	18.33	15	34	27	85	735	675	225	630	139	712	93	275	71	362	4079	3917	161	26.24
6.74	18.64	8	9	<DL	12	135	129	27	114	21	132	19	64	<DL	79	748	719	29	26.34
7.25	21.79	<DL	<DL	<DL	<DL	7	11	<DL	<DL	<DL	16	<DL	14	<DL	<DL	49	49	0	26.79
7.50	23.38	<DL	12	8	13	54	61	12	49	10	62	8	35	<DL	52	373	340	33	26.76
7.60	23.84	<DL	9	<DL	26	107	124	39	124	24	134	18	84	20	103	811	776	35	26.88
Min.		7	9	8	7	7	7	12	8	8	8	8	8	8	7	8	8	0	19.26
Max.		36	84	94	124	778	855	320	1004	213	1140	140	487	104	657	5946	5697	323	28.06
Average		15	35	33	48	240	248	93	254	66	239	47	132	34	167	1402	1298	107	25.72

ACL_22–32_ = (Σ n C_n_ / Σ C_n_); DL = detection limit.

**Table 4:** Molecular distribution of sterols and triterpene diols in ngg^−1^ from sediment core GeoB16202-2.

Depth (m)	Age (ka)	27 Δ^5^	27 Δ^0^	Choles- tanone	28 Δ^5,22^	28 Δ^5^	28 Δ^0^	29 Δ^5,22^	29 Δ^0,22^	29 Δ^5^	29 Δ^0^	30 Δ^22^	C30–1–15 diol	C30–1–15 ketol	C31–1–15 diol	Tara- xerol	b- amirina	Lupeol	Total	29 Δ^0^/29Δ^5^	29 Δ^0.22^/ 29Δ^5.22^	Choles-tanone/27Δ^5^
0.08	1.39	9	<DL	<DL	15	16	8	<DL	<DL	<DL	<DL	<DL	19	8	11	<DL	<DL	<DL	85	–	–	–
0.11	1.57	8	<DL	14	14	15	7	<DL	<DL	<DL	<DL	<DL	10	<DL	7	<DL	<DL	<DL	76	–	–	1.71
0.14	1.85	10	<DL	16	16	<DL	8	<DL	<DL	<DL	<DL	<DL	18	8	10	<DL	<DL	<DL	86	–	–	1.64
0.44	5.65	10	7	16	16	19	<DL	<DL	8	<DL	8	<DL	29	9	10	<DL	<DL	<DL	131	–	–	1.62
0.79	7.47	8	8	14	14	15	7	<DL	10	<DL	10	12	78	18	12	9	<DL	<DL	217	–	–	1.62
0.99	8.36	15	11	24	24	27	13	<DL	10	<DL	10	<DL	22	13	13	85	<DL	<DL	268	–	–	1.65
1.22	9.23	14	13	21	27	24	12	<DL	14	7	14	17	34	15	13	227	<DL	<DL	452	1.85	–	1.48
1.49	10.32	11	11	18	21	19	10	<DL	11	<DL	11	8	98	16	15	483	<DL	<DL	734	–	–	1.68
1.68	11.01	12	18	21	21	23	12	<DL	15	<DL	15	8	41	12	12	256	<DL	<DL	464	–	–	1.77
1.80	11.34	11	9	17	18	19	10	<DL	9	<DL	9	8	55	10	13	57	<DL	<DL	244	–	–	1.52
2.04	11.90	14	29	25	49	17	18	21	65	40	65	153	130	24	25	841	28	21	1565	1.64	3.10	1.79
2.25	12.27	19	16	26	28	30	16	<DL	18	15	18	16	72	25	19	36	<DL	<DL	354	1.20	–	1.33
2.52	12.74	14	8	21	28	23	13	8	23	9	23	34	147	24	33	19	<DL	<DL	428	2.46	2.75	1.54
2.65	12.96	18	39	19	30	20	21	47	69	28	69	116	197	22	24	165	28	23	935	2.43	1.48	1.08
2.78	13.23	9	12	13	22	14	<DL	<DL	23	9	23	22	20	22	8	34	11	<DL	241	2.59	–	1.35
2.98	13.90	13	10	20	19	22	11	<DL	12	8	12	<DL	97	18	21	<DL	<DL	<DL	261	1.59	–	1.54
3.09	14.25	8	<DL	13	15	13	8	<DL	10	<DL	10	12	52	10	11	<DL	<DL	<DL	160	–	–	1.68
3.25	14.63	34	36	25	81	30	27	67	82	138	82	147	137	36	22	46	98	77	1165	0.60	1.23	0.72
3.50	15.14	18	26	24	50	23	17	22	46	45	46	46	151	20	23	47	57	48	709	1.02	2.10	1.29
4.04	16.16	15	15	17	54	18	10	14	26	86	26	64	117	21	21	33	27	37	604	0.31	1.85	1.15
4.40	16.49	12	9	14	17	15	8	9	12	18	12	<DL	95	11	17	<DL	<DL	<DL	249	0.68	1.36	1.16
4.48	16.56	15	14	19	45	22	12	12	23	49	23	67	96	18	21	16	14	15	480	0.46	1.92	1.24
4.60	16.67	26	15	23	34	26	15	24	21	88	21	56	106	21	35	<DL	17	10	539	0.24	0.86	0.86
4.80	16.85	8	7	11	28	11	9	9	19	27	19	32	80	26	19	19	8	11	345	0.70	2.14	1.32
4.98	17.01	17	12	22	35	23	13	9	18	73	18	15	273	38	48	<DL	<DL	<DL	613	0.25	1.94	1.29
5.35	17.32	8	<DL	13	12	14	<DL	<DL	<DL	<DL	<DL	<DL	10	9	11	<DL	<DL	<DL	78	–	–	1.55
5.48	17.42	10	<DL	16	17	19	9	<DL	<DL	9	<DL	<DL	18	8	13	<DL	<DL	<DL	119	–	–	1.59
5.68	17.58	26	20	16	51	21	20	50	95	87	81	105	113	19	27	10	<DL	<DL	742	0.93	1.89	0.62
6.12	17.96	17	20	16	98	19	23	30	90	71	77	109	95	12	26	25	19	22	768	1.07	3.02	0.96
6.30	18.13	19	23	<DL	44	21	25	47	97	82	84	196	133	35	28	19	10	8	871	1.03	2.08	–
6.50	18.33	11	24	21	60	14	20	19	66	30	66	62	33	27	10	12	<DL	8	482	2.20	3.53	1.94
6.74	18.64	14	30	15	23	20	14	16	65	34	65	83	173	35	31	<DL	<DL	<DL	618	1.93	4.21	1.06
7.25	21.79	11	10	17	17	19	10	<DL	15	<DL	15	11	71	16	17	<DL	<DL	<DL	230	–	–	1.48
7.50	23.38	12	17	13	17	15	10	<DL	33	12	33	38	158	39	26	<DL	<DL	<DL	422	2.78	–	1.11
7.60	23.84	9	11	19	20	15	<DL	<DL	10	<DL	10	<DL	12	10	7	<DL	<DL	<DL	122	–	–	2.14
Min.		8	7	11	12	11	7	8	8	7	8	8	10	8	7	9	8	8	76	0.24	0.86	0.62
Max.		34	39	26	98	30	27	67	97	138	84	196	273	39	48	841	98	77	1565	2.78	4.21	2.14
Average		14	17	18	31	19	13	25	34	44	33	58	86	19	19	122	29	26	453	1.33	2.22	1.41

27Δ^5^ = cholesterol, 27Δ^0^= cholestanol, 28Δ^5.22^=diatomsterol, 28Δ^5^=campesterol, 28Δ^0^=campestanol, 29Δ^5.22^=stigmasterol, 29Δ^0.22^=, 29Δ^5^=β-sitosterol, 29Δ^0^= β-sitostanol, dΔ^22^=dinosterol., DL=detection limit.

**Table 5:** Carbon and hydrogen isotopic composition of long-chain n-alkanes (C_29_, C_31_) and fatty acids (C_26_, C_28_) from sediment core GeoB16202–2. Hydrogen data (δ_D_) reported in ‰ VSMOW and carbon data (δ^13^C) reported in ‰ VPDB.

Depth(m)	Age(ka BP)	δD C_26_	δ^13^C_26_	δD C_28_	δ^13^C_28_	δD C_29_	δ^13^C_29_	δD C_31_	δ^13^C_31_
0.08	1.39	−123	−27.5	−116	−27.7	–	−28.6	–	−27.3
0.12	1.57	−128	−27.3	−139	−27.7	−115	−28.3	−127	−27.7
0.36	4.73	−127	−28.5	−128	−29.3	−135*	−29.5*	−143*	−28.5*
0.44	5.65	−136	−27.9	−147	−28.3	−130	−30.0	−142	−29.0
0.55	6.33	−128	−27.9	−129	−28.1	−137*	−29.6*	−145*	−28.8*
0.65	6.77	−129	−29.1	−136	−28.4	−137*	−30.1*	−142*	−29.2*
0.79	7.47	−141	−28.5	−142	−28.8	−144	−30.4	−145	−29.2
0.90	7.99	−123	–	−133	–	−141*	−30.4*	−144*	−29.7*
0.99	8.36	−143	−29.8	−142	−30.7	−141	−31.0	−142	−30.3
1.10	8.79	−140	−30.9	−144	−31.9	−143*	−31.4*	−144*	−30.7*
1.22	9.23	−139	−29.8	−145	−31.1	−147	−31.2	−149	−30.3
1.35	9.71	−143	−30.8	−149	−31.9	−138	–	−141	–
1.49	10.32	−143	−29.9	−139	−30.7	−147	−31.0	−150	−30.1
1.62	10.81	−141	–	−137	–	−140*	−30.7*	−144*	−30.0*
1.68	11.01	−141	−30.0	−147	−30.8	−129	–	−152	–
1.74	11.20	−134	−31.1	−139	−32.7	−146*	−30.6*	−151*	−29.9*
1.80	11.34	−141	−30.4	−148	−31.4	−138	−31.1	−137	−29.9
1.90	11.58	−151	−32.6	−151	−33.7	−142*	−32.4*	−146*	−31.9*
2.04	11.90	−150	−32.1	−151	−33.0	−155	−32.9	−155	−32.4
2.25	12.27	−147	−32.6	−151	−33.8	−140	−33.0	−144	−32.7
2.52	12.74	−148	−32.5	−145	−33.8	−144	−33.1	−144	−32.8
2.65	12.96	−148	−32.4	−152	−33.6	−147	−32.7	−149	−32.4
2.78	13.23	−143	−31.8	−141	−33.1	−149	−32.8	−149	−32.8
2.98	13.90	−134	−31.8	−135	−32.8	−134	−32.2	−136	−32.0
3.09	14.25	−139	−32.1	−142	−32.7	−130	−31.0	−136	−30.5
3.25	14.64	−150	−32.7	−151	−33.2	−139	−32.8	−144	−32.4
3.38	14.90	−157	−33.5	−142	−34.5	−146*	−33.2*	−147*	−32.8*
3.50	15.14	−149	−32.3	−150	−32.8	−145	−32.8	−148	−32.2
3.75	15.63	−149	−33.5	−140	−34.0	−147*	−32.5*	−149*	−31.8*
4.04	16.16	−151	−31.8	−153	−32.3	−159	−32.3	−163	−31.1
4.20	16.31	−157	−32.4	−152	−33.2	−144*	−32.0*	−149*	−30.7*
4.40	16.49	−153	−31.2	−154	−31.5	−148	−31.6	−153	−30.1
4.48	16.56	−153	−31.1	−152	−31.7	−158	−31.7	−163	−30.3
4.60	16.67	−151	−31.1	−150	−31.3	−144	−31.5	−149	−30.2
4.80	16.85	−145	−30.9	−145	−31.4	−145	−31.3	−153	−30.2
4.98	17.01	−148	−30.5	−146	−31.2	−157	−31.4	−162	−29.9
5.35	17.32	−147	−30.3	−143	−31.1	−146	−30.7	−154	−29.5
5.48	17.42	−147	−30.3	−151	−31.0	−156	−30.8	−161	−29.5
5.68	17.58	−145	−30.0	−144	−30.4	−137	−30.2	−143	−29.6
5.90	17.76	−154	–	−146	–	−138	−30.3	−144	−29.2
6.12	17.96	−140	−29.9	−142	−30.2	−141	–	−148	–
6.30	18.13	−141	−29.5	−141	−30.0	−132	−30.0	−139	−29.2
6.50	18.33	−130	−29.4	−131	−30.1	−135	−29.7	−140	−28.6
6.74	18.64	−130	−29.3	−131	−30.4	−141	−30.0	−148	−28.4
7.10	20.80	−138	−30.6	−141	−31.6	−133*	−30.2*	−137*	−29.0*
7.25	21.79	−127	−29.8	−132	−30.6	−130	−30.1	−135	−28.5
7.38	22.62	−130	–	−141	–	−134*	−30.4*	−137*	−28.9*
7.50	23.38	−134	−30.1	−134	−31.2	−125	−30.5	−131	−29.7
7.57	23.74	−143	−30.8	−136	−32.1	−137*	−30.2*	−140*	−29.2*
7.60	23.84	−137	−29.6	−141	−30.3	−126	−29.8	−139	−29.7

Carbon and hydrogen data for fatty acids were corrected using equations previously published [2], [3], [4]. Hydrogen data were corrected considering the change of global volume ice according with [5] and [6]. *Data previously published [7].

## 2 Experimental Design, Materials and Methods

### 2.1 Material and methods

#### 2.1.1 Sampling campaign

A 773 cm-long gravity core GeoB16202-2 (1°54.50′S and 41°35.50′W; 2248 m water depth) (Fig. 1) was retrieved from the continental slope off northeastern Brazil (Barrerinhas / Ceara basin) during RV Maria S. Marian (Cruise 20. Leg 3) expedition in 2012.

#### 2.1.2 Extraction and separation of lipids

The lipid extractions, separation and instrumental analyses (molecular distribution and isotopic composition of carbon (δ^13^C) and hydrogen (δD)) of *n*-alkanes and fatty acids were performed at MARUM-Center for Marine Environmental Science, University of Bremen. The derivatization and analyses of polar fraction were carried out at LabMAM, PUC-Rio. The age model is based on thirteen radiocarbon (^14^C) measurements using planktonic foraminifera specimens (*G. ruber* and *G. sacculifer*). The results were calibrated with the IntCal13 curve (reservoir correction age of 400 ± 200 years) and the age uncertainty was modeled using the software BACON version 2.2 [7].

A total of 35 sediment samples with dry weights between 10 and 15 g were ground for lipid analysis. The extraction of lipids was carried out using a DIONEX Accelerated Solvent Extractor system (ASE 200) with a mixture of dichloromethane: methanol (9:1; v/v) as solvent at 100 °C and 1000 psi for 5 min (three cycles). A standard mixture of squalene, erucic acid and 5α-androstanol was added to each sample prior to extraction as internal standards. After removal of elemental sulfur with copper turnings, the extracts were reduced under a N_2_ flow and were subsequently saponified for 2 h at 85 °C with 1 mL of KOH (0.1 M) in methanol: H_2_O (9:1; v/v). The neutral fractions were recovered with 1 mL of hexane (3 times) and further isolated in a 1% water deactivated silica gel column chromatography (open column of 6 mm X 4 cm) into three sub-fractions (*n*-alkane, ketone and polar lipids) by elution with 4 mL of hexane, dichloromethane and dichloromethane: methanol (1:1; v/v), respectively. The isolated *n-*alkane fraction, was further purified by elution with hexane over 4 cm AgNO_3_-coated silica column to remove unsaturated compounds. The *n-*alkane and ketone fractions were dried under a N_2_ flow and were dissolved in 100 µL of toluene for further analysis of *n*-alkanes and alkenones by gas chromatography with flame ionization detector (GC-FID). The polar compounds in the neutral fraction was derivatized using 100 µL of BSTFA (N.O-bis(trimethylsilyl) trifluoroacetyl acetamide) and 250 µL of acetonitrile with heating at 80 °C for 1 h. The excess of derivatizing reagents were dried out and the residue redissolved in hexane for the determination of sterols and alkanols by gas chromatography-mass spectrometry GC/MS technique. The bulk extract residue after saponification was acidified by addition of two drops of concentrated HCl and the so-called acid fraction extracted three times with 1 mL of hexane/dichloromethane (4:1; v/v)) and then were methylated by the addition of 2 mL of acidified MeOH (with known isotopic composition of −140.8 ± 0.8‰ VSMOW and −55.6 ± 0.7‰ VPDB) with heating at 50 °C for 12 h, to yield fatty acid methyl esters (FAMEs). The FAMEs were recovered from the mixture with 3 × 1 mL of hexane and concentrated down to dryness under a gentle stream of N_2_. The dry extracts were dissolved in dichloromethane and were cleaned by elution with DCM over a column with 0.5 cm of Na_2_SO_4_ and 4 cm of silica gel. The methylated fatty acids extract was transferred to 2 mL vials and was dissolved in 100 µL of toluene prior to determination by GC-FID.

#### 2.1.3 Analyses of lipids by GC-FID and GC–MS

Analyses of *n*-alkanes and fatty acids were performed using a GC-FID system (Focus by Thermo Fisher Scientific) equipped with a capillary column Rxi-5 ms (30 m length X 0.25 mm internal diameter X 0.25 µm film coating) using helium as carrier gas (purity 99.999%) at a constant flow of 1.2 mL min^−1^. The injector temperature was programmed to be constant at 260 °C and injections (1 µL) were made in *splitless* mode with a detector held at 280 °C. For *n*-alkane analyses, the GC oven temperature was set at 60 °C (held for 2 min) increased at 10 °Cmin^−1^ to 150 °C, and then increased at 4 °Cmin^−1^ to 320 °C and held for 12 min. *N*-alkanes were quantified by comparing peak areas of the compounds to external standard mixture (C_19_—C_34_, 10 µg mL^−1^) and to the internal squalene standard. Fatty acids were determined using a GC- oven program from 60 °C (held for 2 min), increased at 6 °C min^−1^ to 300 °C and held for 15 min. Fatty acids were compared with an external standard mixture (C_24_, C_26_, C_28_, C_30_,C_32_ FAMEs, 10 µg mL^−1^) and recovery was evaluated by comparing with the area of erucic acid. Precision of compound quantification is about 5% based on multiple standard analyses.

Analyses of sterols and alkanols were performed on a gas chromatography-mass spectrometry system (GC/MS Trace GC Ultra coupled to DSQ mass spectrometer, Thermo Finnigan) equipped with a DB5-ms capillary column (30 m length X 0.25 mm internal diameter X 0.25 µm film coating), using the following conditions: i) helium as carrier gas (purity 99.999%) at a constant flow of 1.2 mL min^−1^; ii) injector temperature programmed to be constant at 250 °C and injections (1µL) made in *splitless* mode; iii) initial oven temperature at 60 °C, temperature ramp at 20 °C min^−1^ up to 220 °C and then at 2 °C min^−1^ until 300 °C (hold for 15 min); iv) ionization source temperature at 220 °C. The GC/MS system was operated in the electron ionization (EI) mode −70 eV and Full Scan mode (monitoring the range *m/z* = 50–650). For quantification purposes it was used a calibration curve with certificated standards and an internal standard (5α-cholestane). The recovery was evaluated by comparison with the signal of 5α-androstanol added on each sample prior to extraction.

#### 2.1.4 Determination of δ^13^C in lipids by GC-IRMS

Carbon isotope analyses (δ^13^C) of individual compounds (*n*-alkanes and fatty acids) were performed on a gas chromatograph Trace GC Ultra, coupled to a Finnigan MAT 252 mass spectrometer (IRMS, Thermo Scientific) via a combustion interface operated at 1000 °C. Into CG-IRMS system the lipid extract is injected in *splitless* mode, the compounds of interest are separated by a capillary column and they are oxidized to CO_2_ by a combustion reactor at 1000 °C. This CO_2_ produced is injected into mass spectrometer where it is analyzed. Separation of n-alkanes and fatty acids were made using a HP-5 ms capillary column (30 m length X 0.25 mm internal diameter X 0.25 µm film coating), with helium as carrier gas (purity 99.999%), at a constant flow of 1.5 mL min^−1^. The injector temperature was programmed to be constant at 250 °C and the injections (2µL) were done in *splitless* mode. The GC temperature, for n-alkane analyses, was programmed from 120 °C (hold time 3 min), followed by heating at 5 °C min^−1^ to 320 °C (hold time 15 min). The oven program for fatty acids analyses was 30 °C min^−1^ to 170 °C, 2 °C min^−1^ to 215 °C (hold time of 12 min), and 20 °C min^−1^ to 240 °C (hold time of 15 min). The δ^13^C values for individual compounds were calibrated by injecting pulses of CO_2_ as a reference gas that was automatically introduced into IRMS at the beginning and end of each analysis. The δ^13^C data is reported in per mil (‰) relative to the VPDB standard. Samples were analyzed in duplicate and the standard mixture of *n*-alkanes was injected every six determinations. The reported δ^13^C values represent an average of duplicates with a standard deviation less than 0.5‰. The formation of fatty acid methyl ester from the fatty acid involved the addition of one methanol carbon with known isotopic composition per fatty acid molecule. The δ^13^C ratio for methanol, used to derivatize the fatty acids, was determined (−55.6 ± 0.7‰VPDB) with an elemental analyzer (EA-Flash, Thermo Scientific) to perform the correction of the isotopic compositions in methylated samples. The isotopic carbon composition of fatty acids was corrected according to published equations [2,3], as shown below.

$$\delta^{13}C_{\text{FA}}=\left[\left(C_{n+1}\right)\delta^{13}C_{\text{FAME}}-\delta^{13}C_{\text{MeOH}}\right]/C_{n}$$

#### 2.1.5 Determination of δD in lipids by GC-IRMS

The δD compositions of fatty acids and n-alkanes were measured on a Thermo Trace GC, equipped with a HP-5 ms column (30 m length X 0.25 mm internal diameter X 1.0 µm film coating), coupled to a Thermo Fisher MAT 253 (IRMS) via a pyrolysis reactor (operated at 1420 °C). The GC oven program was similar to the conditions used for the analysis of the carbon isotopic composition (δ^13^C). The measurement accuracy was controlled by the injection of standards of known isotopic composition after every six measurements and by the daily determination of the H_3_^+^ factor using H_2_ as the reference gas. The δD values were calibrated by the external H_2_ reference gas, are reported in ‰ VSMOW, and were corrected considering the effect of changes in the global ice volume [5]. All samples were analyzed in duplicate and the reported values represent the mean with a standard deviation less than 3‰. The δD of fatty acids was corrected considering the addition of a methyl group according to published equations [2,4], as shown below.

$$\left(H_{n}+3\right)\delta D_{\text{FAME}}=H_{n}\delta D_{\text{FA}}+H_{n}+3\delta D_{\text{MeO}}$$

## Declaration of Competing Interest

The authors declare that they have no known competing financial interests or personal relationships which have, or could be perceived to have, influenced the work reported in this article.

## Appendix Supplementary Materials

Image, application 1
